# Gemstone Spectral CT Virtual Noncontrast Images and Iodine Maps for the Characterization of Thyroid Lesions and Distinguishing Thyroid Papillary Carcinoma from Nodular Goiter

**DOI:** 10.1155/2023/8220034

**Published:** 2023-02-27

**Authors:** Chun Yao, Xiaofeng Chen, Zhiqi Yang, Ruibin Huang, Sheng Zhang, Yuting Liao, Xiangguang Chen, Zhuozhi Dai

**Affiliations:** ^1^Department of Radiology, Meizhou People's Hospital, Meizhou 514031, China; ^2^Guangdong Provincial Key Laboratory of Precision Medicine and Clinical Translational Research of Hakka Population, Meizhou 514031, China; ^3^Department of Radiology, The First Affiliated Hospital of Shantou University Medical College, Shantou 515000, China; ^4^GE Healthcare, Guangzhou 510623, China; ^5^Department of Radiology, Shantou Central Hospital, Shantou, Guangdong 515031, China; ^6^Department of Radiology, Sun Yat-Sen Memorial Hospital, Sun Yat-Sen University, Guangzhou, Guangdong 510120, China

## Abstract

**Background:**

Gemstone spectral contrast-enhanced CT with virtual noncontrast (VNC) images and iodine maps can potentially reduce the number of required CT scans for thyroid lesions. However, data regarding the clinical utility of VNC images and iodine maps in characterizing thyroid lesions and distinguishing thyroid papillary carcinoma from nodular goiter are still limited.

**Purpose:**

To determine whether VNC images and iodine density could reliably aid in characterizing thyroid lesions and distinguishing thyroid papillary carcinoma from nodular goiter compared with true noncontrast (TNC) images.

**Methods:**

This retrospective study included patients with thyroid papillary carcinoma or nodular goiter who underwent TNC and contrast-enhanced gemstone spectral CT scans. The consistency of qualitative parameters, including intralesional calcification, necrosis, lesion boundary, thyroid edge interruption, and lymph node metastasis, between TNC and VNC images, was analyzed using the kappa statistic. TNC attenuation, VNC attenuation, absolute attenuation between TNC and VNC, and iodine density were compared between thyroid papillary carcinoma and nodular goiter by using Student's *t*-test. The diagnostic performance for distinguishing papillary carcinoma from nodular goiter was evaluated by using the area under the receiver operating characteristic curve (AUC) value, sensitivity, and specificity.

**Results:**

VNC and TNC imaging showed comparable performance in delineating calcification, necrosis, lesion boundary, thyroid edge interruption, and lymph node metastasis (all *k* > 0.75). Papillary carcinoma showed significantly lower absolute attenuation between VNC and TNC than nodular goiter (7.86 ± 6.74 vs. 13.43 ± 10.53, *P*=0.026), which was similarly observed for iodine density (31.45 ± 8.51 vs. 37.27 ± 10.34, *P*=0.016). The iodine density showed higher diagnostic performance (AUC = 0.727), accuracy (0.773 vs. 0.667), sensitivity (0.750 vs. 0.708), and specificity (0.786 vs. 0.643) than the absolute attenuation between TNC and VNC images (AUC = 0.683).

**Conclusions:**

VNC imaging, a promising substitute for TNC imaging, has comparable diagnostic efficacy for reliably characterizing thyroid lesions. Iodine density could be valuable for distinguishing thyroid papillary carcinoma from nodular goiter.

## 1. Background

Thyroid nodules are commonly observed in more than half of adult patients receiving thyroid imaging examinations, approximately 7% of which are malignant. The accurate diagnosis of thyroid carcinoma is a challenge for isolated thyroid nodules with significant enhancement of the solid component and without small calcifications, obvious necrosis/cystic areas, thyroid edge interruption signs, or lymph node metastasis in computed tomography (CT) images [1–4]. As the majority of thyroid nodules are asymptomatic, accurate diagnosis, and differentiation before initiating definitive treatment are vital so that patients can avoid undergoing unnecessary invasive procedures [3, 4].

Fine needle aspiration biopsy has been promoted as the most effective method, with high sensitivity and specificity [5]. However, its application is routinely advised against for suspected cases of malignancy due to its invasiveness. Ultrasound is the preferred imaging modality for the assessment of thyroid nodules, and it has a good diagnostic ability to detect morphological features and cystic necrosis of thyroid nodules [6]. However, ultrasound may has limited in evaluating invasion to adjacent structures, evaluation of lymph nodes at low cervical levels (such as levels VI and upper mediastina), substernal goiter, and thyroid nodules with coarse calcifications that may reduce the quality of ultrasound image of its internal and surrounding structure [7]. In addition, previous studies have shown that ultrasound has a relatively low diagnostic performance in differentiating between benign and malignant thyroid nodules [8, 9].

As an alternative approach, CT, including unenhanced and contrast-enhanced examinations, has been proven to be a useful complementary examination for the characterization of thyroid lesions, especially to evaluate invasion to adjacent structures, the cervical lymph node metastasis, substernal goiter and intranodular coarse calcification [10–13]. In addition, CT has advantages in the classification of incidental thyroid nodules, since incidental thyroid nodules are regularly found in CT scans [14, 15]. However, CT has some limitations in thyroid nodules because of radiosensitivity (especially in children) and a higher cost [4, 16, 17] than ultrasonography, which does not have radiation hazards and is more affordable. Thus, effectively reducing the CT radiation dose while meeting diagnostic requirements is one of the primary objectives of radiation protection [18], especially in radiation-sensitive organs.

The advanced technology of gemstone spectral CT, equipped with the material-suppressed iodine technique, can support material separation in contrast-enhanced CT images and has the advantages of optimizing the inspection procedure, shortening the inspection time, and reducing the radiation dose [19–21], which have gained increasing clinical interest. Recent studies have indicated that VNC could evaluate lesions in a sophisticated manner, identifying hypoattenuating pancreatic injury, urinary stones, and intracranial hemorrhage, and could significantly reduce the radiation dose to the patient [22, 23]. Although VNC technology has been gradually applied to the chest and abdominal examinations, its application in the neck, especially in thyroid disease, remains to be explored [24, 25]. True noncontrast (TNC) image is a standardized diagnostic method recognized clinically for thyroid nodules. However, limited data are available regarding the clinical utility of VNC images and their potential to replace TNC images for characterizing thyroid nodules and distinguishing thyroid papillary carcinoma from nodular goiter.

Therefore, this study aimed to determine whether VNC images can reliably characterize thyroid lesions, reduce the effective radiation dose in thyroid diseases, distinguish thyroid papillary carcinoma from nodular goiter compared with TNC images, and explore its potential to replace TNC images.

## 2. Methods

### 2.1. Patients

**Figure 1:**
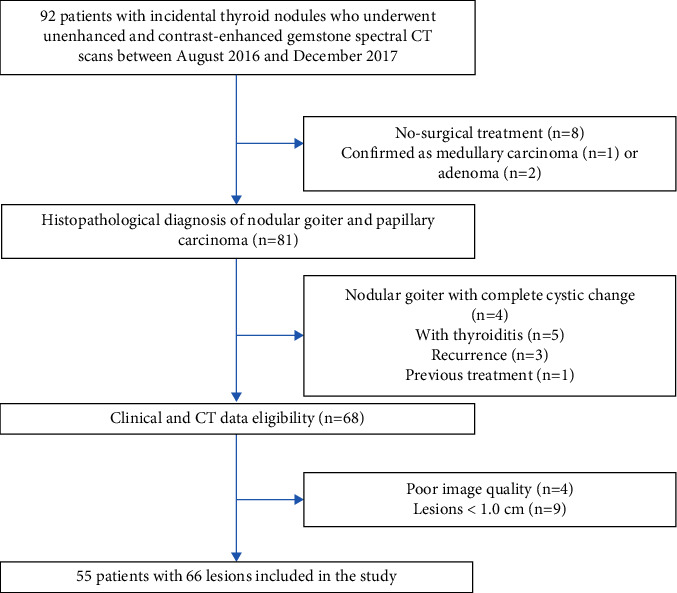
Study flowchart for the inclusion and exclusion of patients to obtain the final patient cohort.

This study was approved by the Institutional Review Board, and the requirement for written informed consent from patients was waived. A population database consisting of 92 patients with incidental thyroid nodules who underwent unenhanced and contrast-enhanced gemstone spectral CT scans between August 2016 and December 2017 was queried to identify patients suffering from thyroid papillary carcinoma or nodular goiter confirmed by postoperative histopathology. Thyroid adenomas and medullary carcinoma were excluded due to obviously cystic change and collarbone artifacts on lesions. A total of 81 patients were ultimately eligible for inclusion. The following 26 patients were excluded: (1) disease recurrence, (2) nodular goiter with complete cystic change, (3) previous treatment, (4) with thyroiditis, (5) lesions <1.0 cm [20], and (6) poor image quality. Finally, 55 GS patients were included in this study (Figure 1).

### 2.2. Image Acquisition

All patients underwent triphasic CT scanning on gemstone spectral CT (GE, Discovery HD750), including a TNC scan, a routine arterial contrast-enhanced scan, and a gemstone spectral venous contrast-enhanced scan. The scanning range was from the basis cranii to the upper edge of the aortic arch, according to the recommended thyroid scanning protocol in our institution. During the scanning, the patients were asked to raise the inferior jaw without swallowing and lower the shoulders to avoid clavicle interference. TNC and routine arterial contrast-enhanced scans were both performed with the following scan parameters: tube voltage: 120 kVp, automatic tube current modulation (auto mA), pitch: 0.984, rotation time: 0.8 s, detector coverage: 40.0 mm, slice thickness: 0.625 mm, and slice interval: 0.625 mm. The parameters used for the subsequent gemstone spectral venous contrast-enhanced scan were as follows: fast kV switching between 80 and 140 kVp, tube current: 260 mA, rotation time: 0.8 s, pixel spacing: 0.625 mm, field of view: 25 cm × 25 cm, pitch: 0.984, slice thickness: 5.0 mm, and slice interval: 5.0 mm. After the intravenous injection of contrast medium (3.0–3.5 ml/s, 1.5 ml/kg, Omnipaque, 350 mg·I/ml, GE) via a syringe pump (Bayer Healthcare), routine arterial contrast-enhanced and gemstone spectral venous contrast-enhanced were performed with delays of 30 s and 60 s, respectively. Adaptive statistical iterative reconstruction and automatic tube current modulation techniques were used for dose reduction. Axial images with a slice thickness and slice interval of 1.25 mm were reconstructed. In each scan, the volume CT dose index (CTDIvol, mGy) and dose-length product (DLP, mGy.cm) for each scan were obtained directly from the dosimetry metrics displayed in the CT scanner to estimate the radiation dose.

### 2.3. Image Analysis

All data were sent to an ADW 4.6 workstation (GE). The venous-phase VNC images were automatically produced by gemstone spectral imaging (GSI) material-suppressed iodine software after processing. The thyroid nodules were identified on TNC images with the area of lower attenuation corresponding to the nonhomogeneous enhancement regions on contrast-enhanced CT images according to the surgical records. The quantitative and qualitative features of VNC and TNC images were evaluated with GSI volume viewer software and volume rendering software, respectively. The measurement window width and level were 350 HU and 40 HU, respectively, for both the VNC and TNC images.

For the qualitative analysis, two radiologists (C.Y. and Z.Y., with 11 and 13 years of experience, respectively) without previous knowledge of the diagnoses made using TNC images and VNC images, independently characterized the thyroid lesions two weeks after the first evaluation. Their characterization was recorded, including the following: boundary of the lesion (0, clear; 1, unclear), thyroid edge interruption (0, absent; 1, present), intralesional calcification (0, negative; 1, positive), necrosis (0, negative; 1, positive), and capability of lymph node metastasis (0, negative; 1, positive). Lymph node metastasis was deemed present if at least one of the following CT conditions was observed [17]: heterogeneous enhancement, intralesional calcification, or necrotic or cystic changes. Thyroid edge interruption was defined as the disappearance of the high-density boundary of the thyroid.

The attenuation of lesions in the VNC and TNC images and the iodine density of lesions in the iodine maps were evaluated by the two aforementioned senior radiologists who were blinded to the clinical and laboratory findings. By manually placing regions of interest (ROIs) with an area of 10.0–25.0 mm^2^ on the largest slice of the lesion with the most abundant solid components of lesions, the attenuation of lesions in the TNC images and VNC images and the iodine density of lesions in iodine maps were recorded, with obvious areas of necrosis, calcification, artifacts, and cystic areas being avoided. The largest nodules was measured for multiple nodules. The transverse diameter and anterior-posterior diameter were also recorded. All measurements were performed three times, and then the results were averaged for further analysis. The iodine/VNC rate was defined as iodine density divided by VNC attenuation [20]. Absolute attenuation was defined as the absolute difference in attenuation between VNC and TNC images [20]. The findings were verified by another senior radiologist (X.C., with 16 years of experience) if disagreement occurred.

### 2.4. Statistical Analysis

All statistical analyses were performed using R software (version 3.6.4; https://www.r-project.org/). A two-tailed *P* value <0.05 indicated a statistically significant difference. The patient characteristics and radiation dose were compared with Student's *t*-test (for continuous variables) and a chi-squared test (for nominal variables). The attenuation comparison between TNC images and VNC images and the comparison of quantitative parameters between nodular goiter and papillary carcinoma were performed using Student's *t*-test. The consistency of qualitative and quantitative parameters between two observers was analyzed with Cohen's kappa statistic and the interclass correlation coefficient (ICC), while that between TNC images and VNC images was analyzed using the kappa statistic, Pearson's correlation coefficient, and a Bland–Altman analysis. A receiver operating characteristic curve (ROC) analysis was performed to differentiate papillary carcinoma from nodular goiter, and the optimal cutoff value of quantitative parameters was determined with the Youden index. The area under the curve (AUC), accuracy, sensitivity, and specificity were also calculated.

To validate the reproducibility of quantitative and qualitative parameters, the tumor measurement was completed by observers 1 and 2 separately for all 55 patients with 66 lesions and 28 patients with 35 lesions who were randomly selected from the whole cohort to assess the interobserver consistency [26].

## 3. Results

### 3.1. Patient Characteristics and Radiation Doses

In total, 55 patients with 66 lesions (mean age: 47 years; range: 21–75 years) were included. Forty-four patients had unilateral thyroid lesions (32 cases of thyroid papillary carcinoma and 12 cases of nodular goiter), and 11 patients had bilateral thyroid lesions (4 cases of bilateral papillary carcinoma, 5 cases of bilateral nodular goiter, and 2 cases of unilateral papillary carcinoma and unilateral nodular goiter). The characteristics and radiation doses of all patients are shown in Table 1.

### 3.2. Interobserver Consistency of Quantitative and Qualitative Parameters

The interobserver consistency of qualitative parameters between the two observers was consistent (all *k* > 0.75). The ICC values for the attenuation on TNC images and VNC images were 0.95 and 0.89 between the two observers, respectively.

### 3.3. Comparison between VNC Images and TNC Images

**Figure 2:**
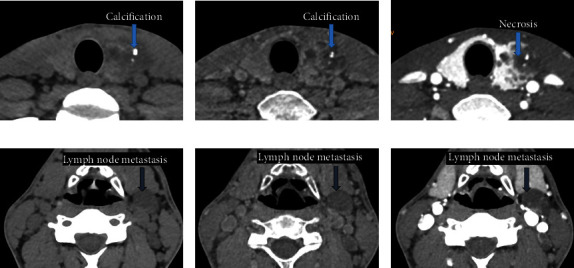
Thyroid CT images from a patient with papillary thyroid carcinoma. Compared with true noncontrast (TNC) images (a), virtual noncontrast (VNC) images (b) had a clear boundary of thyroid lesions. In displaying calcification, necrosis, and lymph node metastasis, VNC images (b and e) were consistent with TNC images (a and d). There was no enhancement of necrosis in the contrast-enhanced images (c and f).

For qualitative image analysis, our data showed good overall consistency between the VNC images and TNC images with respect to the characterization of thyroid lesions (all *k* > 0.75; Table 2). Compared with TNC images, the average characterization error rates of thyroid lesions with VNC images were 7.58% (5/66), 1.51% (1/66), 12.1% (8/66), and 1.51% (1/66) for calcification, necrosis, lesion boundary, and detection capability of lymph node metastasis, respectively. Representative VNC and TNC images are presented in Figure 2.

The quantitative image analysis data showed a moderate positive correlation in the attenuation between VNC images and TNC images (Table 3), and there was a significant difference observed for papillary carcinoma (*r* = 0.47; *P* < 0.001), but no difference for nodular goiter (*r* = 0.33; *P*=0.116). Furthermore, there were no significant difference in the CT attenuation between VNC images and TNC images for papillary carcinoma (*P*=0.093) and nodular goiter (*P*=0.053).

**Figure 3:**
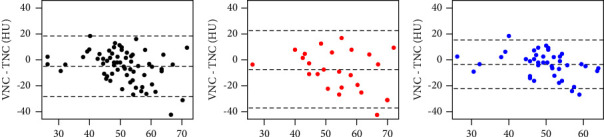
Bland–Altman plots show the agreement between true noncontrast (TNC) attenuation and virtual noncontrast (VNC) attenuation in all lesions (a), nodular goiter (b), and papillary carcinoma (c).

Further comparison between the papillary carcinoma group and nodular goiter group revealed no significant difference in either VNC or TNC attenuation (*P*=0.078 and *P*=0.400, respectively; Table 4). The absolute attenuation between VNC images and TNC images was 9.89 HU ± 8.67 for all thyroid lesions. A higher absolute attenuation between VNC images and TNC images was also found for the nodular goiter group than for the papillary carcinoma group (13.43 ± 10.53 vs. 7.86 ± 6.74; *P*=0.026). Moreover, iodine density was higher in the nodular goiter group than in the papillary carcinoma group (37.27 ± 10.34 vs. 31.45 ± 8.51, *P*=0.016). The Bland–Altman plots showed a mean bias of −4.97 HU (95% CI: −28.92–18.99), −7.58 HU (95% CI: −37.90–22.73), and −3.47 HU (95% CI: −22.71–15.77) for all lesions, nodular goiter, and papillary carcinoma, respectively (Figure 3).

### 3.4. Diagnostic Performance for Papillary Carcinoma

**Figure 4:**
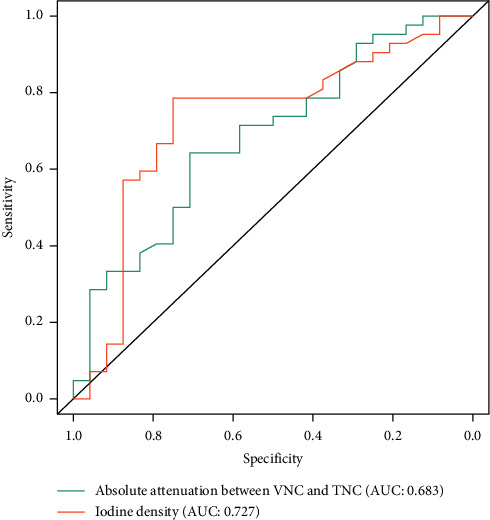
Receiver operating characteristic curve of the absolute attenuation between virtual noncontrast (VNC) and true noncontrast (TNC) images and iodine density for distinguishing papillary carcinoma from nodular goiter.

**Figure 5:**
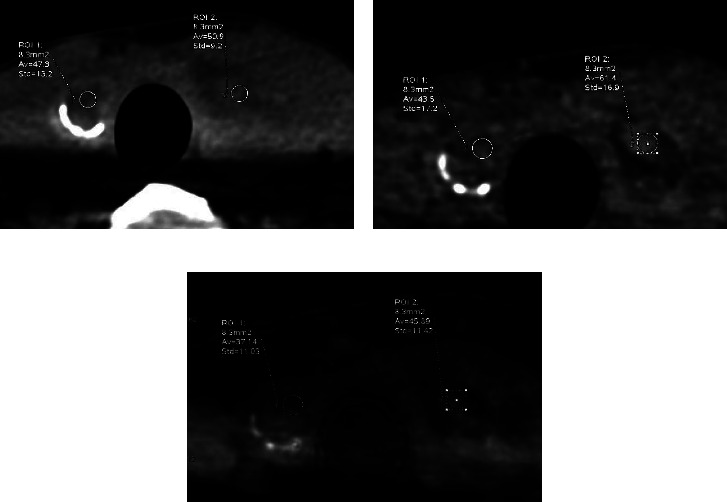
Axial true noncontrast (TNC) and venous-phase virtual noncontrast images (VNC) from a 44-year-old woman with papillary carcinoma (right side) and nodular goiter (left side). The absolute attenuation between TNC (a) and VNC (b) images for papillary carcinoma (3.8 HU) was smaller than that for nodular goiter (10.5 HU). The iodine density (c) of nodular goiter (left side) was higher than that of papillary carcinoma (right side) (45.89 vs. 37.14; cutoff = 38.35).

Figure 4 presents the ROC of absolute attenuation between VNC and TNC and iodine density for differentiating papillary carcinoma from nodular goiter. Compared with the absolute attenuation between VNC and TNC, iodine density showed a higher performance (AUC: 0.727 vs. 0.683), with higher accuracy (0.773 vs. 0.667), sensitivity (0.750 vs. 0.708), and specificity (0.786 vs. 0.643). The optimal cutoff value was 7.55 HU and 38.35 (100 *μ*g/cm^3^), as calculated with the Youden index of absolute attenuation between VNC and TNC images and iodine density for differentiating papillary carcinoma from nodular goiter, respectively. Representative VNC, TNC, and iodine density images of papillary carcinoma and nodular goiter are presented in Figure 5.

## 4. Discussion

Our study demonstrates that VNC imaging is comparable to TNC imaging in terms of diagnostic efficacy. The ability of VNC images to display calcification, necrosis, lesion boundary, thyroid edge interruption, and lymph node metastasis is comparable to that of TNC images. The kappa analysis showed that these two techniques have good consistency (*k* > 0.75), which is concordant with recent VNC studies [20, 21, 27–29]. These results suggest that VNC imaging can be a potential substitute for TNC imaging in characterizing thyroid nodules [27–29]. Furthermore, our study showed that papillary carcinoma had a significantly lower absolute attenuation between VNC and TNC than nodular goiter (7.86 ± 6.74 vs. 13.43 ± 10.53; *P*=0.026), which was similarly observed in the iodine density (31.45 ± 8.51 vs. 37.27 ± 10.34; *P*=0.016). In addition, iodine density is a valuable parameter for distinguishing thyroid papillary carcinoma from nodular goiter, which may enlarge the utility of CT in thyroid nodules.

Similar to previous studies, our study demonstrates that VNC imaging is comparable with TNC imaging in terms of diagnostic efficacy. The ability of VNC images to display calcification, necrosis, lesion boundary, and lymph node metastasis is comparable with that of TNC images, which is consistent with recent VNC studies [20, 21, 27–29]. Intranodular calcification is one of the important imaging characteristics for the differential diagnosis of thyroid nodules. In our study, the average characterization error rate of thyroid lesions on VNC images for calcification compared with TNC images was 7.58% (5/66). A similar result was demonstrated in study indicating that the measured mean percent error for calcification on the revolution CT was 10.0% [30]. A potential explanation for the results may be as follows: the VNC technique may have a certain decalcification effect on these characterizations under iodine suppression on the enhanced images [29]. Regarding intralesional necrosis, lesion boundary, and lymph node metastasis, the average characterization error rate on VNC images compared with TNC images was 1.51% (1/66), 12.1% (8/66), and 1.51% (1/66), respectively. Similar findings were noted in previous reports [20, 21, 27, 28]. The aforementioned error rates may be related to the size, density, composition of lesions, and CT partial volume effect but may be acceptable for thyroid lesions in terms of avoiding unnecessary additional costs and radiation burden from repeat imaging [20]. Furthermore, the agreement analysis demonstrated a moderate positive correlation in the attenuation between VNC images and TNC images. This observation is partially consistent with the data from previous studies, which showed a good correlation between VNC and TNC attenuations of various anatomic regions, with differences <10 HU [20, 21, 27, 28, 31–33]. This difference may be caused by multiple factors, including different patient cohorts with different contrast media phases, patient sizes, various DECT systems, and subjective image interpretations.

It has been reported that the iodine concentration in the venous phase could be used to discriminate benign and malignant lesions using DECT, such as renal lesions, adrenal adenomas versus metastasis, or solitary pulmonary lesions. Lee et al. used DECT iodine quantification to classify the focal thyroid lesions and found that the iodine concentration value was highest in benign nodules and lower in papillary thyroid carcinoma [13]. Zegadło et al. demonstrated that the concentration of iodine in malignant tumors was significantly higher than that of benign lesions in the arterial phase and venous phase using a single-source DECT, which may have practical use in the differential diagnosis of solitary pulmonary nodules [34]. Nagayama et al. analyzed the DECT results in patients with adrenal adenomas or metastases and showed that iodine density during the portal venous phase was higher in adenomas than in metastasis [20]. Although the thyroid gland has different effects on iodine uptake and utilization than other organs, our findings are similar to those in other sites. For instance, iodine density in the portal venous phase was higher in nodular goiter than in papillary carcinoma (37.27 ± 10.34 vs. 31.45 ± 8.51; *P* = 0.016), enabling discrimination, which is consistent with previous reports [13, 35–37]. The underlying differences in pathology provide an explanation for this: a benign nodule is formed due to follicular epithelial hyperplasia and uneven recovery, with fiber septum formation or colloid storage. This means that the normal epithelium with the function of iodine uptake is present. However, the epithelium is disrupted in a malignant nodule and replaced by prominent papillae or other neoplastic cells [13, 35–37].

Consistent with a previous study, our data showed that the simple measurement of CT attenuation does not yet allow for this type of prompt differential diagnosis [20], with an AUC of 0.683 and accuracy of 0.667 for differential diagnosis, suggesting that a combination of VNC and contrast images is still necessary for thyroid diagnosis. The iodine density from contrast images showed a higher performance (AUC: 0.727 vs. 0.683) than the absolute attenuation between VNC images and TNC images in differentiating papillary carcinoma from nodular goiter, with higher accuracy (0.773 vs. 0.667), sensitivity (0.750 vs. 0.708), and specificity (0.786 vs. 0.643). Our results, however, are not superior to those of Li et al. [37], who reported 0.643∼0.929 sensitivity and 0.794∼0.936 specificity in detecting papillary carcinoma using iodine quantification. This discrepancy may be attributed to the small sample size and differences in scan protocols and injection protocols.

Our study has several limitations. First, this was a retrospective study performed at one institution. Therefore, the results of this study should be validated in a multicenter study. Second, lesions <1.0 cm was excluded from this study, which may limit the clinical application of TNC image. Third, this is a relatively small cohort study and the results should be validated in a lager large cohort study. Finally, we only enrolled patients with papillary carcinoma (the most common type of thyroid cancer); thus, our results may not be universally applicable to other thyroid malignancies, such as follicular, medullary, or anaplastic thyroid carcinoma.

## 5. Conclusions

In conclusion, the results of this study demonstrate that the diagnostic efficacy of VNC imaging is similar to that of TNC imaging, allowing for the reliable characterization of thyroid lesions. Thus, it is a promising substitute for TNC imaging. Iodine density could be a valuable parameter for distinguishing thyroid papillary carcinoma from nodular goiter.

## Figures and Tables

**Table 1:** Patient characteristics and radiation dose.

Variable	Total number of lesions (*n* = 66)	Nodular goiter (*n* = 24)	Papillary carcinoma (*n* = 42)	*P* value
Patient age (y)	47.18 ± 15.17	55.96 ± 10.50	42.17 ± 15.23	<0.001^a^
Male (y)	42.44 ± 18.83	58.57 ± 10.83	29.89 ± 13.11	<0.001^a^
Female (y)	48.70 ± 13.67	54.88 ± 10.51	45.52 ± 14.15	0.020^a^
Sex^*∗*^				
Male	11 (16.67%)	3 (12.50%)	8 (19.05%)	0.731^b^
Female	55 (83.33%)	21 (87.50%)	34 (80.95%)	
Transverse diameter (cm)				
TNC	2.62 ± 1.58	3.29 ± 1.80	2.24 ± 1.32	0.009^a^
VNC	2.66 ± 1.55	3.32 ± 1.68	2.28 ± 1.35	0.008^a^
Anterior-posterior diameter (cm)				
TNC	2.06 ± 1.30	2.72 ± 1.52	1.68 ± 0.98	0.005^a^
VNC	2.08 ± 1.26	2.77 ± 1.47	1.69 ± 0.92	0.003^a^
CTDIvol (mGy)				
TNC	15.51 ± 2.19	15.21 ± 2.42	15.68 ± 2.06	0.405^a^
Routine arterial phase	21.76 ± 2.28	21.59 ± 2.82	21.86 ± 1.94	0.685^a^
GSI venous phase	10.30 ± 0.00	10.30 ± 0.00	10.30 ± 0.00	0.999^a^
DLP (mGy.cm)				
TNC	403.81 ± 86.19	390.14 ± 86.38	411.63 ± 86.14	0.334^a^
Routine arterial phase	569.74 ± 71.81	556.97 ± 83.81	577.04 ± 63.90	0.316^a^
GSI venous phase	267.50 ± 32.19	269.78 ± 31.99	266.20 ± 32.62	0.667^a^
Thyroid lesions^*∗*^				0.030^b^
Unilateral	44 (66.67%)	12 (50.00%)	32 (76.19%)	
Bilateral	22 (33.33%)	12 (50.00%)	10 (23.81%)	

*Note.* ^*∗*^ The results are the measurements with a corresponding ratio in the parentheses, and the remainder of the results is mean ± standard deviation. *P*^a^: student's *t*-test and *P*^b^: chi-squared test. TNC: true noncontrast, VNC: virtual noncontrast, CTDIvol: volume CT dose index, and DLP: dose-length product.

**Table 2:** Qualitative analysis of the characterization of thyroid lesions between VNC images and TNC images.

	VNC (*n* = 66)	TNC (*n* = 66)	*k*	*P* value
Calcification			0.85	<0.001
Negative	35 (53.03%)	30 (45.45%)		
Positive	31 (46.97%)	36 (54.55%)		
Necrosis			0.96	<0.001
Negative	47 (71.21%)	48 (72.73%)		
Positive	19 (28.79%)	18 (27.27%)		
Lesion boundary			0.76	<0.001
Clear	36 (54.55%)	42 (63.64%)		
Unclear	30 (45.45%)	24 (36.36%)		
Thyroid edge interruption			1.00	<0.001
Absent	30 (45.45%)	30 (45.45%)		
Present	36 (54.55%)	36 (54.55%)		
Lymph node metastasis			0.94	<0.001
Negative	57 (86.36%)	56 (84.85%)		
Positive	9 (13.64%)	10 (15.15%)		

Note. *k* and *P* values were computed with the kappa statistic. TNC: true noncontrast and VNC: virtual noncontrast.

**Table 3:** Quantitative analysis of the attenuation of thyroid lesions between VNC and TNC images.

	VNC	TNC	*r* ^a^ (95 CI%)	*P* ^a^ value	*P* ^b^ value
Attenuation (HU)					
Papillary carcinoma	51.02 ± 10.69	47.55 ± 7.79	0.47 (0.20, 0.68)	<0.001	0.093
Nodular goiter	57.33 ± 14.99	49.75 ± 11.18	0.33 (−0.08, 0.65)	0.116	0.053

Note. ^a^*r* and corresponding *P*^a^ value were computed using Spearman's correlation test; *P*^b^ value was computed using student's *t*-test. TNC: true noncontrast and VNC: virtual noncontrast.

**Table 4:** Quantitative analysis of TNC and VNC between papillary carcinoma and nodular goiter.

Variable	Papillary carcinoma	Nodular goiter	*P* ^b^ value
TNC attenuation (HU)	51.02 ± 10.69	57.33 ± 14.99	0.078
VNC attenuation (HU)	47.55 ± 7.79	49.75 ± 11.18	0.400
Absolute attenuation (HU)	7.86 ± 6.74	13.43 ± 10.53	0.026
Iodine density (100 *μ*g/cm^3^)	31.45 ± 8.51	37.27 ± 10.34	0.016
Iodine/VNC ratio	0.68 ± 0.27	0.76 ± 0.24	0.242

Note. *P*^b^: student's *t*-test. TNC: true noncontrast and VNC: virtual noncontrast.

## Data Availability

The data cohorts used and/or analyzed in the present study are available from the corresponding authors upon reasonable request.

## Abbreviations

DECT: Dual-energy CT
VNC: Virtual noncontrast
TNC: True noncontrast
AUC: Area under the curve
GSI: Gemstone spectral imaging.
